# Effect of Accelerated Degradation on the Dimensions and Mechanical Performance of 3D-Printed PLA Parts Using Different Filament Manufacturing Techniques

**DOI:** 10.3390/ma18102267

**Published:** 2025-05-13

**Authors:** Laura Castanon-Jano, Mario Lozano-Corona, Elena Blanco-Fernandez

**Affiliations:** 1 Area of Manufacturing Processes, School of Industrial Engineering and Telecommunications, Universidad de Cantabria, 39005 Santander, Spain; mario.lozano@unican.es; 2 GITECO Research Group, School of Civil Engineering, Universidad de Cantabria, 39005 Santander, Spain; elena.blanco@unican.es

**Keywords:** formworks, 3D printing, polymers, degradation, metal, additive, UV, alkaline pH

## Abstract

Polymer 3D printing is popular due to its accessibility and low material waste. While commonly used in prototyping and medical applications, its potential for molds in complex concrete geometries, such as heritage reproductions or artificial reefs, remains underexplored. These applications require resistance to degradation from UV exposure, rain, and highly alkaline concrete (pH~13). This study evaluates the accelerated degradation of 3D-printed PLA specimens. Four PLA types were tested: virgin PLA extruded in the lab, commercial PLA, PLA with 50% metal powder, and PLA with encapsulated metal powder. Rectangular specimens were printed and tested under flexural loads following ISO-167 standards. Initially, their performance was assessed without exposure. Then, half of the specimens underwent UV and rain simulation, while the rest were immersed in an alkaline solution (pH 13, 50 °C). Dimensional changes and flexural strength were measured at five intervals. Exposure to an alkaline medium at 50 °C is more aggressive than UV radiation, limiting the lifespan of PLA formwork. Adding metal powder weakens PLA by 65% after 7 days, making it unsuitable. Printing defects accelerate degradation. Unmodified PLA is the best choice for concrete formwork, with commercial PLA and PLA from pellets showing nearly identical behavior.

## 1. Introduction

In recent years, additive manufacturing has experienced a remarkable growth, establishing itself as a key technology in several industries [1,2,3,4]. Among the many techniques available, Fused Deposition Modeling (FDM) stands out for its accessibility and versatility. This method is based on the extrusion of thermoplastic filaments layer by layer for the fabrication of three-dimensional objects, making it an ideal choice for rapid prototyping and customized production.

One of the sectors where FDM printing is becoming increasingly relevant is the construction industry [5,6]. In particular, 3D-printed formwork manufacturing has emerged as an innovative alternative to traditional methods. To date, formwork has mainly been made of wood or metal panels made of steel or aluminum. While the former are economical but have limited durability, the latter offer greater strength and flexibility in terms of design. However, both have a common limitation: the difficulty in generating complex shapes, such as curved geometries, organic designs, or elements with recesses. In this context, FDM printing offers a promising solution by enabling the fast and cost-effective production of formwork with a significant reduction in waste and greater freedom in the design of complex shapes.

**Figure 1:**
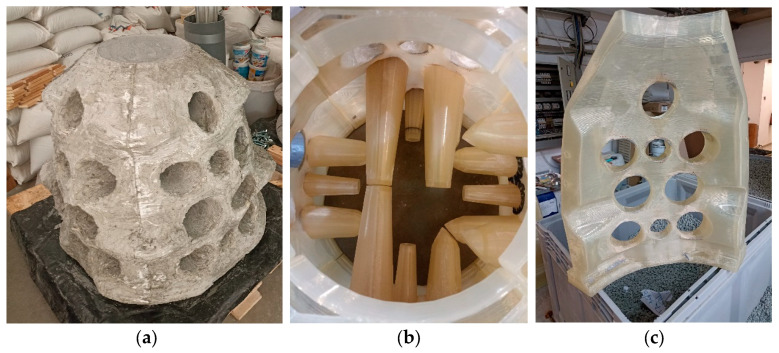
Concrete artificial reef manufactured using 3D-printed formwork: (**a**) final concrete piece, (**b**) PLA formwork cores for hole generation, and (**c**) one of the four PLA formwork walls.

Since 2016, several studies have explored the application of this technology in the manufacture of structural and decorative elements, such as stairs [5], domes and columns [6], ornamental components [6,7], and even artificial reefs (Figure 1). In the latter, various 3D printing-related manufacturing approaches [8] and materials [9] have been reported, although this work will be focused on their production using 3D printing formworks used to cast the final mortar-based geometry. After curing, they are submerged in the sea. For all applications involving formworks—whether structural, decorative, or for artificial reef construction—reusable molds have been employed in some cases. In others, particularly when producing unique geometries, skin-type formworks, for only one use, are preferred in order to minimize material consumption [10].

While the results have been promising, the implementation of 3D-printed formwork using FDM poses certain challenges, especially with regard to its integrity and durability. In particular, reusable formworks must withstand not only direct contact with concrete but also the adverse environmental conditions to which they may be exposed during use and storage. As thermoplastics have a variable sensitivity to these factors, it is essential to assess their performance in real construction environments.

One of the most critical exposure conditions for polymeric formworks occurs during the casting and early curing stages of concrete. In these circumstances, the fresh mortar remains in prolonged contact with the formwork under highly alkaline conditions, typically with a pH around 13. Despite the relevance of this environment for the intended application, the available literature is limited and often not directly applicable. Some existing studies have explored the effects of alkaline pH on PLA-based materials [11], but often under conditions that do not accurately replicate the continuous and immersive exposure typical of concrete casting processes. In this case, accelerated degradation with a drop of concentrated sodium hydroxide is applied to the sample, and the degradation is assessed almost immediately, with exposure times of just 1 min for bending tests and 10 min for creep tests.

Another example is [12], which investigates PLA degradation in alkaline media, but in the context of replacing traditional steel reinforcement with 3D-printed PLA lattice structures. In such studies, the mechanical performance is derived from concrete specimens with embedded PLA rather than from the polymer material itself after exposure. This makes it difficult to isolate and understand the degradation behavior of the PLA component alone, and even more so to extrapolate the results to its application as a reusable formwork.

Furthermore, the limited number of studies that do examine PLA degradation tend to focus primarily on chemical characterization [13], with minimal attention paid to the evolution of mechanical properties, dimensional stability, or mass loss over time—factors that are critical for the integrity and reusability of formwork structures. Among those that do consider printed specimens [14], most neglect the combined mechanical and geometrical effects that occur under alkaline exposure.

In the specific context of reusable formworks, these properties are paramount. The polymeric elements must maintain their strength to withstand mechanical fastening (via straps or screws), resist hydrostatic pressure exerted by the fresh concrete, and endure demolding forces once the concrete has cured. Any significant degradation of mechanical strength or volumetric shrinkage not only compromises the reusability of the formwork but also affects the dimensional accuracy and surface quality of the resulting concrete component.

For these reasons, it is essential to conduct targeted degradation tests under controlled alkaline conditions, representative of actual casting environments, in order to evaluate the feasibility of PLA-based materials for this application

The second environmental condition considered in this study corresponds to the exposure of the material to atmospheric agents, primarily sunlight and rain, which are inherently accompanied by variations in temperature and humidity. In this context, the existing literature on the environmental degradation of PLA parts is limited and highly heterogeneous in terms of both methodology and findings. There is no consensus on maximum exposure thresholds or degradation kinetics for PLA under real outdoor conditions.

Previous studies have explored various degradation setups, ranging from controlled temperature and humidity chambers without solar radiation [15] to artificial UV radiation exposure using 1000 W halogen lamps [16] and even unmonitored open-air aging [17]. The diversity in test conditions—including exposure times, radiation intensities, and the presence or absence of moisture—leads to widely varying outcomes. Reported effects range from slight increases in mechanical strength under dry UV exposure [16] to significant strength reductions of up to 30% after prolonged outdoor weathering. However, these results are difficult to interpret or extrapolate, as critical environmental parameters such as UV intensity, precipitation frequency, and temperature fluctuations are often unknown or poorly controlled.

Furthermore, it must be considered that climatic conditions vary significantly depending on geographic location, which further limits the generalizability of existing data. Therefore, to reliably assess the suitability of PLA-based materials for reusable formwork in construction applications, it becomes essential to simulate UV radiation and rain exposure under controlled laboratory conditions. This allows for reproducible and quantifiable evaluation of the material’s degradation behavior, providing insights into its long-term performance in outdoor environments.

An essential aspect in the fabrication of polymeric formworks for mortar casting is the appropriate selection of the polymeric material. In this context, the most commonly employed materials in Fused Deposition Modeling (FDM) for construction-related applications are PLA, PETg, and TPU [18,19]. Among them, PLA has gained particular attention due to its low cost and excellent printability. Furthermore, PLA is a highly valued material as it is derived from renewable resources such as corn starch, sugarcane, or sugar beet. Since its production does not rely on fossil-based feedstocks, it contributes to a lower carbon footprint and is considered a more sustainable alternative in terms of greenhouse gas emissions and energy consumption throughout its life cycle.

In recent years, there has been a growing availability of PLA-based filaments incorporating various additives, such as metals, wood, or textile fibers [20,21,22,23,24,25], which can enhance certain material properties compared to neat PLA. These improvements may include increased mechanical strength [26], thermal conductivity [27], or other functional characteristics. However, to date, no PLA-based composite filament has been specifically investigated for its suitability in formwork fabrication. Therefore, building on the advantages of PLA as a base material, this study explores the incorporation of an industrial by-product as an additive. This approach aims not only to reduce the amount of plastic required but also to valorize a secondary material from another production process. Furthermore, the method by which the polymer matrix (PLA) and additives are combined plays a critical role in determining the mechanical strength and dimensional stability of the resulting filament material [28,29]. It is, therefore, necessary to evaluate whether the direct blending of PLA pellets with the powdered additive yields a homogeneous filament upon extrusion or if an alternative mixing strategy must be identified to enhance the uniformity and overall quality of the composite.

The performance of the additive-enhanced materials must be benchmarked against that of virgin PLA. In this context, two material formats are considered: commercially available PLA filament and PLA in pellet form. Although both are expected to exhibit comparable intrinsic properties, information obtained through consultations with manufacturers indicates that the production of PLA filaments—particularly those with color additives—often involves the incorporation of plasticizers. These additives are introduced to enhance certain mechanical characteristics, such as reducing stiffness and brittleness. As a result, it becomes necessary to systematically assess potential differences in the mechanical response of both material formats, particularly under pre- and post-degradation conditions. This comparison will focus on PLA filaments containing colorants versus untreated PLA pellets. Furthermore, given the increasing adoption of large-format 3D printing systems that frequently utilize pellet-fed extruders, it is critical to evaluate whether the feedstock form and extrusion method significantly affect the overall material behavior and structural performance.

The novelty of the present research lies in its comprehensive and application-driven approach to evaluating the suitability of PLA-based materials—including novel PLA composites reinforced with industrial by-products—for use as reusable 3D-printed formwork in mortar casting. Unlike previous studies, which typically focus on isolated degradation mechanisms or neglect the influence of printing and processing variables, this work systematically investigates the impact of realistic alkaline and outdoor exposure conditions on the mechanical integrity and dimensional stability of printed PLA specimens. Moreover, this study compares different material formulations and delivery formats (filament vs. pellet-based extrusion), providing new insights into their performance and feasibility in large-format FDM printing for the construction sector. This integrated methodology aims to establish robust criteria for material selection and processing strategies, ultimately supporting the development of more sustainable and durable polymeric formworks for complex concrete geometries.

## 2. Materials

The materials used in this study can be divided into two main categories: those employed in the fabrication of the specimens subjected to degradation and evaluation and auxiliary substances necessary for establishing the degradation conditions.

### 2.1. Polymeric Materials

Three polymeric compositions were selected for the fabrication of the test specimens. All of them are based on polylactic acid (PLA), a biodegradable and compostable thermoplastic polymer derived from renewable resources, such as corn starch or sugarcane. PLA is widely used in 3D printing due to its ease of processing, cost effectiveness, and favorable mechanical and dimensional stability under standard conditions. Although other polymers may offer superior mechanical performance, they are often more expensive and environmentally harmful upon degradation.

Given the large quantity of material required for the fabrication of reusable formworks, it is necessary to evaluate whether PLA can provide sufficient durability or if degradation will rapidly compromise its structural integrity. While it is known that PLA undergoes hydrolysis as a natural degradation mechanism, previous studies (references to be added) have indicated that at ambient temperature and under neutral pH conditions, the loss of performance occurs slowly. These findings support the hypothesis that PLA could be a suitable candidate for reusable formworks in concrete casting.

Two different PLA formats were selected to evaluate potential differences in degradation behavior related to formulation and processing. The first is a commercial gray PLA filament (1.75 mm diameter) supplied by EOLAS Prints (Cantabria, Spain). The second consists of transparent PLA pellets from the same brand. The use of both formats allows for the assessment of the effect of common additives—such as plasticizers or flow modifiers—present in commercial filaments, which may influence thermal and mechanical behavior. The PLA pellets were processed in-house using a single-screw extruder, ensuring that the resulting specimens contained no additives beyond the polymer base, thus enabling a controlled comparison.

### 2.2. PLA–Metal Composites

To reduce the overall plastic content of the formworks and align with growing environmental concerns regarding plastic usage, a composition was explored by incorporating metal powder into the PLA matrix. The selected powder, commonly referred to as scale, is a by-product of the steel rolling process supplied by the CELSA Group. It originates from the oxidation of molten steel upon exposure to air, forming a brittle oxide layer that detaches during rolling. The resulting powder is primarily composed of iron (≈97%) along with small amounts of manganese, copper, zinc, and other oxides (Table 1). This reuse of industrial by-products not only contributes to waste reduction but also enables the investigation of the influence of such fillers on the degradation behavior of the composite. Although the mechanical reinforcement offered by metal powders is often limited, in some cases, it has shown marginal improvements or trade-offs in performance [21]. This additive was considered potentially compatible with alkaline environments, as metallic reinforcement is commonly used in cementitious systems. Although limited evidence exists regarding the behavior of metal-filled polymers under such conditions, the use of an iron-based scale was hypothesized to offer improved performance if properly dispersed and was, therefore, evaluated using two different mixing approaches.

### 2.3. Degradation Agents

To simulate the degradation processes likely to affect PLA-based formworks in real applications, two agents were used: distilled water and a 40 wt% aqueous solution of sodium hydroxide (NaOH). These simulate the alkaline and humid conditions generated by contact with fresh mortar during casting. Exposure to these agents is expected to replicate the early-stage chemical and environmental stressors acting on the formwork, including the alkaline pH and moisture retention characteristic of fresh concrete, as well as potential storage conditions under atmospheric exposure (e.g., sunlight and rain).

## 3. Methods

### 3.1. Case Studies

The materials described in Section 2 were used and combined to generate 4 types of specimens, which differ in their composition and method of filament manufacture.

Type 1: PLAco

♦ Composition: PLA supplied by the manufacturer in filament form, with gray coloring.
♦ Filament manufacturing method: this filament is manufactured on the company’s premises using an industrial single-screw extruder with water cooling and subsequent winding. The raw material consists of PLA pellets combined with a small quantity of pellets that have the gray pigmentation that will give the filament its final color. These pigmented pellets could include some plasticizer components to improve their mechanical performance, but this is not specified in the datasheet.

Type 2: PLApe

♦ Composition: transparent PLA pellets
♦ Filament manufacturing method: this filament is manufactured with a desktop extruder available in our department, which has air ventilation.

Type 3: PLAmet_trad

♦ Composition: 48.5% by weight of transparent PLA pellets, 48.5% by weight of metal powder, and 3% by weight of coconut oil.
♦ Filament manufacturing method: the pellets are impregnated with coconut oil and mixed with the metal powder in a container, which is poured into the hopper of the desktop extruder. The oil helps the powder to stick to the pellets and not fall to the bottom of the hopper, avoiding as far as possible an uneven composition of the filament along its entire length.

Type 4: PLAmet_caps

♦ Composition: 50% by weight of transparent PLA pellets and 50% by weight of metal powder.
♦ Filament manufacturing method: in order to achieve total homogeneity in the composition of the filament, small capsules are printed with filament made from PLA pellets, which are filled with metal powder and then closed. These capsules are the ones that feed the extruder hopper to print PLA filament additive with metal powder with a more regular composition.

Forty-five specimens of each of the four typologies were produced. Therefore, a total of 180 specimens were produced. Of the 45 specimens in each case, 20 were subjected to degradation by atmospheric agents (UVA + spraying), 20 to degradation by basic pH and hydrolysis, and 5 were left undegraded in order to be able to carry out a comparative analysis with the intact material (Table 2).

The 20 degraded specimens were divided into 5 groups of 4 specimens, which were subjected to different degradation times in order to see the evolution of degradation over time. The degradation times are also detailed in the following sections and depend on the type of degradation and the composition of the material.

### 3.2. Stages of Specimen Manufacture

**Figure 2:**
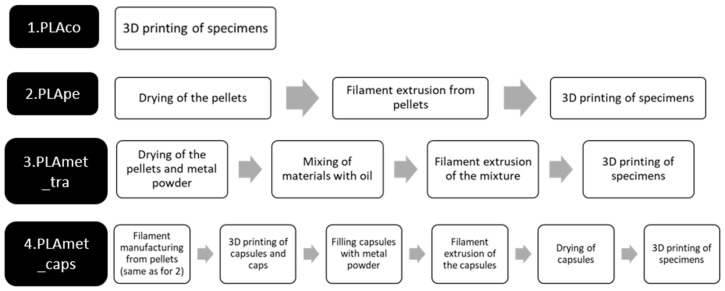
Process flow for the four case studies.

The manufacture of the specimens was carried out differently, depending on the type of specimen. Figure 2 summarizes the manufacturing stages in chronological order for each of the 4 types of specimens.

The pellets, metal, and capsules are dried in an Indelab forced ventilation oven at a temperature of 60 °C for 24 h in order to eliminate the moisture present in the materials and thus facilitate the stabilization of the filament diameter during the extrusion process.

The extrusion of the filament to obtain the PLApe, PLAmet_tra, and PLAmet_caps test pieces is carried out using the 3devo Composer 450 desktop extruder (Utrecht, The Netherlands), equipped with an extrusion screw and designed to create filaments from additivated materials and mixtures, thanks to its fluted screw at the end. The material is fed through the hopper, passes through the extrusion screw, which has 4 independent heating zones, and then exits through the nozzle, where it is cooled by the action of two fans and wound. The temperatures used in the heating elements are shown in Table 3.

The capsules and test tubes were printed on the Artillery Sidewinder X1 3D printer (Artillery 3D Technology Co., Ltd., Shenzhen, China). The slicer used was the free Ultimaker Cura software 5.7.0. The geometry of the standardized capsules and specimens was created in the CAD software Inventor 2024 (Autodesk, San Francisco, CA, USA). The files were saved in .stl format and imported into Cura, where the printing parameters, detailed in Table 4, were configured.

**Figure 3:**
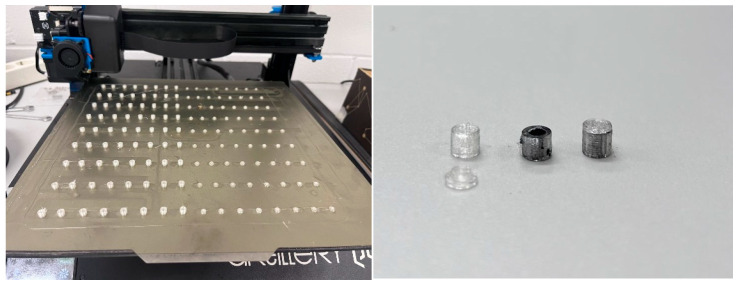
Printing the capsules on the Artillery Sidewinder X1 printer (**left**) and encapsulation stages (**right**): empty, filled, and closed with a lid.

In the case of the capsules, the entire printing bed was filled with bases and lids (Figure 3) in order to reduce the final manufacturing time. For the PLA samples, they were printed in batches of 4, while the PLA samples with metal in their two versions, given the greater complexity of obtaining the filament and the need to avoid wasting it in case of failed prints, were printed one at a time.

### 3.3. Degradation Under Atmospheric Conditions

**Figure 4:**
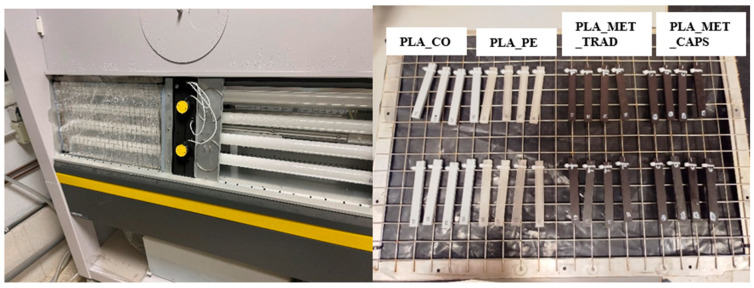
UV test equipment and arrangement of the test tubes on the metal grids.

In order to simulate the atmospheric conditions to which a formwork may be exposed during use or outdoor storage, the specimens are placed in the ATLAS UVTest^®^ Fluorescent/UV Instrument (Mount Prospect, IL, USA) (Figure 4). Due to the lack of specific standards on atmospheric degradation for 3D-printed plastics, the decision was made to apply the existing standards for generic polymers [30]. The machine operates in 6 h cycles, with 5 h of exposure to UVA radiation with an intensity of 0.77 W/m^2^ (simulating solar exposure) and 1 h of distilled water spraying (trying to reproduce exposure to rain). All the specimens were placed in a metal grid (Figure 2), and 16 specimens, 4 of each type, were extracted every 14 days. Therefore, the maximum exposure of the last extraction is 70 days, equivalent to 10 weeks.

### 3.4. Alkaline pH Degradation

**Figure 5:**
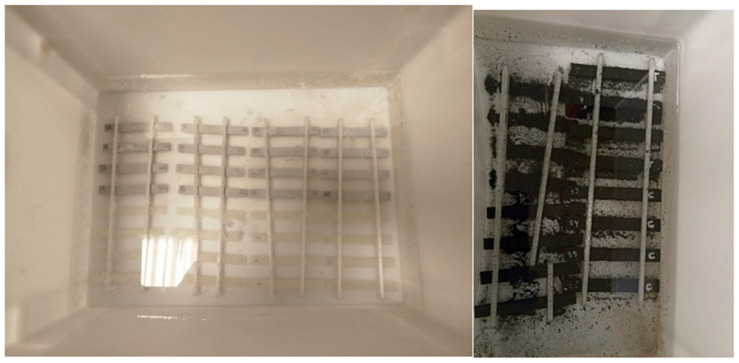
Exposure of PLA (**left**) and PLA+metal (**right**) specimens in basic pH.

Fresh concrete has two characteristics that could progressively deteriorate the PLA formwork. Firstly, there is the water, which could infiltrate between the layers or between passes of the same layer, and secondly, fresh concrete has a basic pH of approximately 13. In addition, the curing of the concrete causes an exothermic reaction, so that temperatures of up to 50 °C can be reached, depending on the volume of concrete being formed. This temperature accelerates the degradation effect on PLA. In order to try to reproduce these conditions, the test specimens are immersed in a solution of NaOH (caustic soda) in water, at a rate of 50 g of soda in 5 L of water (Figure 5). After making 3 solutions with the same water/soda ratio and subsequent measurement with a pH meter, the pH obtained is 12.7. To ensure that the pH remained stable and representative of a highly alkaline environment, the solution (composed of water and sodium hydroxide) was renewed every 7 days, following findings in the literature indicating that PLA degradation tends to neutralize the pH over time [31]. After a series of preliminary tests, it is found that the PLA+metal samples suffer a more accelerated degradation than those of pure PLA and that those manufactured in the traditional way are the most damaged. Therefore, different extraction periods are established for the specimens from the solution for each case, which means different exposure times, although this has been necessary so that the specimens most susceptible to this exposure do not disintegrate completely. Five extractions of each type are carried out as follows:

♦ PLA_co: extractions every 7 days;
♦ PLA_pe: extractions every 7 days;
♦ PLAmet_tra: extracted after exposures of 1 day, 3 days, 5 days, 7 days, and 11 days;
♦ PLAmet_caps: extracted after exposures of 3 days, 7 days, 11 days, 14 days, and 21 days.

### 3.5. Measurement of Dimensional Changes in Specimens

In order to evaluate the variations in the dimensions and weight of the specimens after exposure to the degradation processes, it is essential to measure and weigh each specimen both before and after exposure. This procedure allows the changes experienced by the specimens to be accurately analyzed. A high-precision balance is used to record the weight. Dimensional measurements (length × width × height) are made using a precision caliper.

### 3.6. Flexural Tests

The bending tests were carried out in accordance with UNE-EN-ISO 178:2019 [32]. These tests were carried out using the Zwick Roell Z100 universal testing machine (Ulm, Germany). The specimens are prismatic in shape, with a length of 80 mm, a width of 10 mm, and a height of 4 mm. In the test, the supports are spaced 64 mm apart. A point load was applied to the center of the specimen and was applied by displacement control at a rate of 2 mm/min.

### 3.7. 3D Scanning

**Figure 6:**
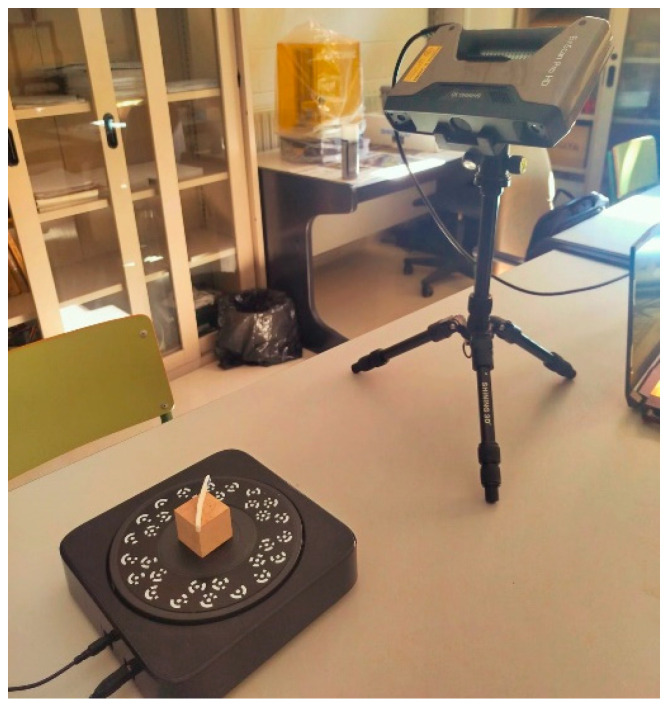
Fixed scanning with a rotating platform of the EinScan Pro HD scanner.

For a more detailed analysis of some of the cases, a representative specimen was selected for each of the degradation processes corresponding to the four materials analyzed. These specimens were subjected to 3D scanning, which allowed a detailed record of deformations and dimensional variations to be obtained. This procedure provides a more accurate assessment of the impact of degradation on each type of material. The scanner used is the Einscan Pro HD, from the company Shining 3D (Hangzhou, China) (Figure 6). It is a structured light scanner. Using a beam of light, the scanner is able to calculate the distance from the emitting point to a point on an object within the range of its trajectory. The point cloud generated in the scan defines the geometry of the three-dimensional object under study. This scanner has an accuracy of 0.04 mm when performing a fixed scan (scanner placed on a tripod) with a turntable, as shown in Figure 6. 

## 4. Results and Discussion

### 4.1. Undegraded Material

**Figure 7:**
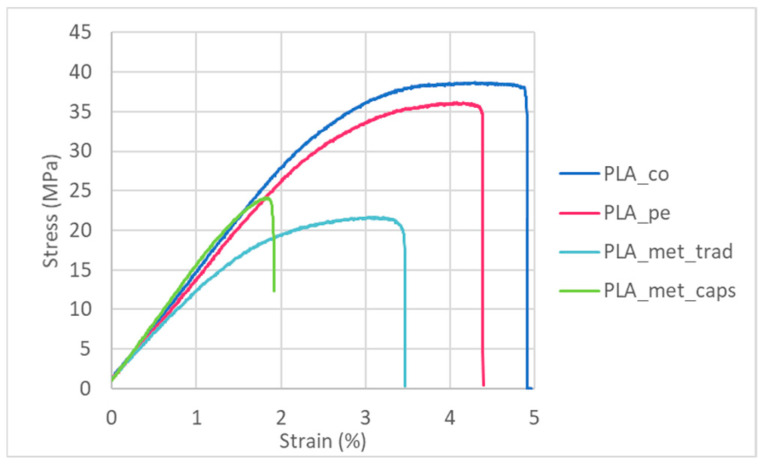
Representative stress–strain curves corresponding to bending tests previous to any degradation.

**Figure 8:**
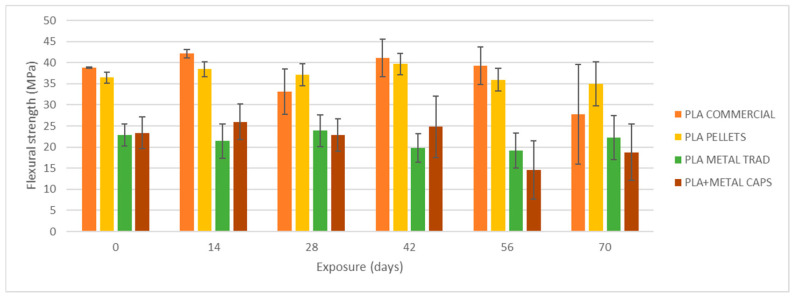
Variation of flexural strength with UVA+ water spray exposure.

**Figure 9:**
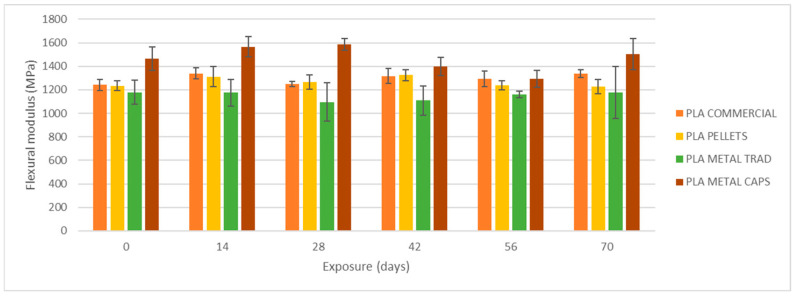
Variation of elastic flexural modulus with UVA+ water spray exposure.

**Figure 10:**
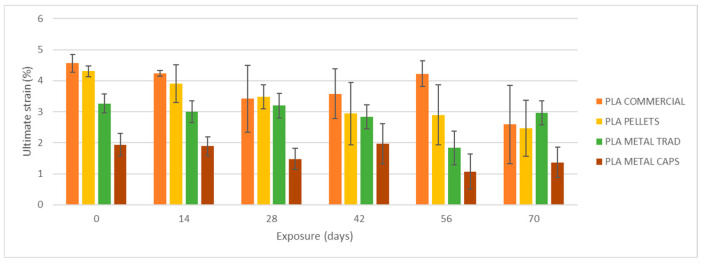
Variation of strain at break with UVA+ water spray exposure.

To begin, the mechanical properties of the materials have been evaluated without degradation, i.e., after an exposure of 0 days (Figure 7). On the left side in Figure 8, Figure 9 and Figure 10, the results of maximum flexural strength, bending modulus of elasticity, and strain at break are presented. In this initial state, the elastic modulus of the commercial PLA and pellet PLA specimens is almost identical. The main difference between the two materials lies in the maximum flexural strength and strain at break, with these values being slightly lower for PLA from pellets (reductions of approximately 2 MPa and 0.2%).

When PLA is combined with metal powder, more variability in the results is observed, depending on the manufacturing method used. In particular, direct mixing of PLA pellets with metal powder results in a more elastic and deformable material, while the composite obtained by the encapsulation technique shows a significantly higher stiffness (increase of about 24%) and a 41% reduction in its deformation capacity. As for the flexural strength, the values obtained in both cases with metal are comparable to each other but show a 39.8% decrease compared to the commercial PLA supplied in filament form. This behavior is clearly seen in Figure 7, where one representative curve of each type is represented. Although mechanical testing was performed on five non-degraded specimens for each material, only one representative stress–strain curve is shown in order to enhance the clarity of the comparison between different materials. This curve was selected as it reflects the typical behavior observed across all tested samples.

The differences observed in the elastic modulus and strain at break can be attributed to the manufacturing method used. In the case of direct mixing of PLA and metal powder (PLA_met_trad), there is a slight loss of powder during the process due to its adhesion to the walls of the mixing vessel or the feed hopper. As a consequence, the fraction of metal incorporated into the filament is slightly lower. On the other hand, the use of capsules allows us to retain all the metal powder during the manufacturing process, avoiding losses and generating a material with a higher charge content, which contributes to an increase in its stiffness and brittleness. In addition, a minimal proportion of oil is incorporated in the direct mixing, which could favor the deformability of the material by acting as a lubricant between the powder particles and the PLA matrix.

### 4.2. UVA Exposure and Water Spraying

Figure 8, Figure 9 and Figure 10 show the evolution of the flexural strength, the elastic flexural modulus, and the flexural strain at the break of the specimens subjected to environmental conditions of combined exposure to ultraviolet radiation and water spray (UVA + spray).

The results indicate that neither the flexural strength nor the elastic modulus shows significant variations in the commercial PLA specimens or those made from pellets after exposure. However, a reduction in the strain at break is observed, especially after 70 days of exposure. In addition, the longer the exposure time, the greater the increase in the dispersion of the results for all four types of specimens, as reflected by the deviation bars in the three graphs. This suggests that UV and spray-induced degradation leads to less predictable mechanical behavior, resulting in a higher variability in the flexural strength values at failure.

**Figure 11:**
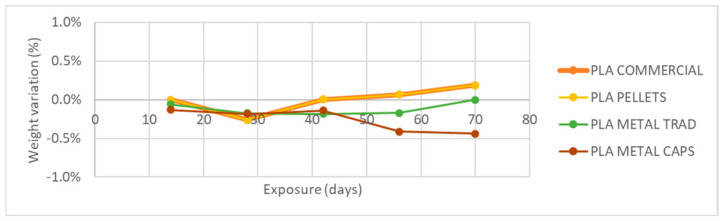
Variation of specimen weight with UVA+ water spray exposure.

The analysis of the weight variation (Figure 11) reveals that none of the four case studies shows significant changes after exposure to atmospheric agents. The recorded variations do not exceed 0.5% in any case and can, therefore, be considered negligible.

**Figure 12:**
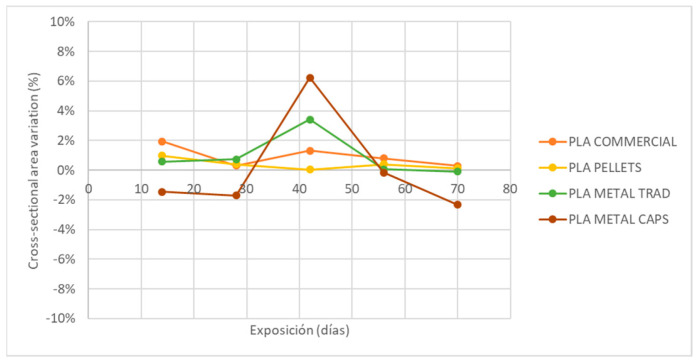
Variation of specimen cross-section with UVA+ spray exposure.

Finally, Figure 12 shows the variation in the cross-sectional area of the specimens after exposure. In all cases, except for the metal PLA specimens made by encapsulation, the variation in area remains between 0 and 3.4%, suggesting some degree of swelling during exposure.

However, in the specific case of metal PLA obtained by encapsulation, a reduction in the cross-section is observed after all exposures, with the exception of the third extraction (45 days), where an increase in area is recorded. Despite this erratic behavior, it is not considered to be relevant because its variation does not exceed 6% in any case. In order to better understand the causes of this phenomenon, a microscopic analysis of the samples by scanning electron microscopy (SEM) is proposed as a future work.

As shown in Figure 8, Figure 9, Figure 10, Figure 11 and Figure 12, the differences observed in the four evaluated parameters (flexural strength, flexural modulus, ultimate strain, cross-sectional change, and mass change) are relatively small. To support the interpretation of these results and to assess whether the variations caused by UV exposure and spraying are statistically significant, an ANOVA (Analysis of Variance) was performed using a significance level of α = 0.05. If the *p*-value is less than or equal to the significance level, the null hypothesis is rejected, indicating that not all population means are equal. Table 5 summarizes all evaluated parameters along with their corresponding *p*-values.

No statistically significant differences were identified in flexural strength or flexural modulus across the four tested materials. Although this trend was already apparent from the graphical analysis (Figure 8 and Figure 9), it is now quantitatively confirmed through inferential statistics. Conversely, ultimate strain exhibited a statistically significant response to the applied degradation conditions. As for the weight and cross-sectional changes, statistical significance was not uniform and appeared to be material dependent; in the case of non-additivated materials (commercial PLA and PLA pellets), UV and spraying exposure did not induce statistically significant variations in mass.

**Figure 13:**
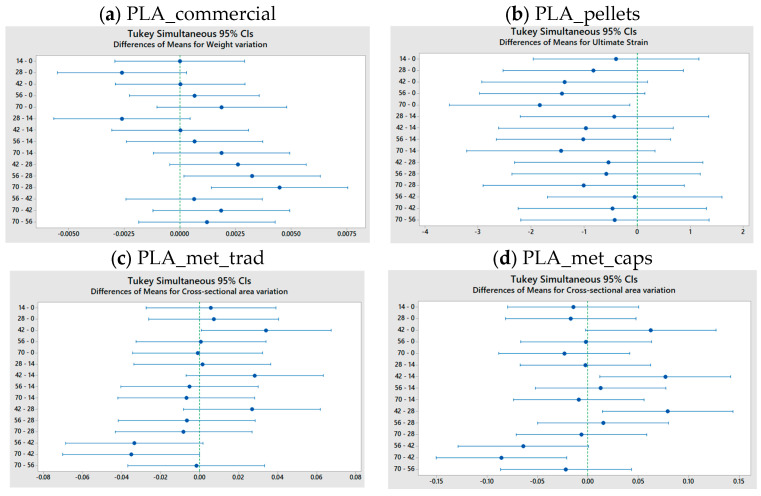
Tukey graphs analyzing pairs of group means on (**a**) weight reduction on PLA_commercial, (**b**) ultimate strain on PLA_pellets, (**c**) cross-sectional area variation on PLA_met_trad, and (**d**) cross-sectional area variation on PLA_met_caps.

For those response variables where the ANOVA indicated a significant overall effect, a Tukey’s Honest Significant Difference (HSD) post hoc test was performed. The most relevant pairwise comparisons are depicted in the plots in Figure 13. These allow for the identification of specific exposure durations whose group means differ significantly. In this analysis, the groups correspond to the exposure times (0, 14, 28, 42, 56, and 60 days). Confidence intervals that do not intersect the zero line indicate statistically significant differences between the corresponding group means.

Figure 13a reveals that the global statistical significance observed for weight reduction in PLA_commercial is primarily driven by significant differences between the exposure durations of 28 and 56 days, as well as between 28 and 70 days. In the case of PLA_pellets (Figure 13b), the overall significant difference arises mainly from the contrast between the means at 0 and 70 days of exposure. However, the results lie very close to the threshold of statistical significance, as is also the case in Figure 13c, where the confidence intervals for the significant pairwise comparisons barely deviate from the vertical zero line. Finally, for PLA_met_caps (Figure 13d), the *p*-value is considerably lower, and the corresponding plot clearly shows statistically significant differences across three exposure groups, with confidence intervals that deviate substantially from zero, indicating a more pronounced degradation effect.

### 4.3. Exposure to Basic pH

**Figure 14:**
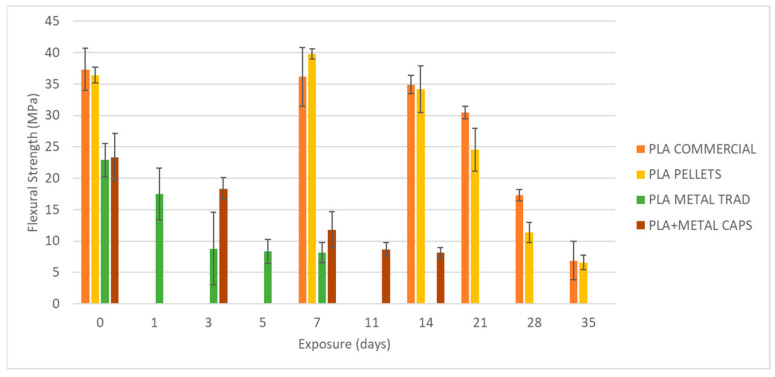
Variation of flexural strength with exposure to basic pH and temperature.

**Figure 15:**
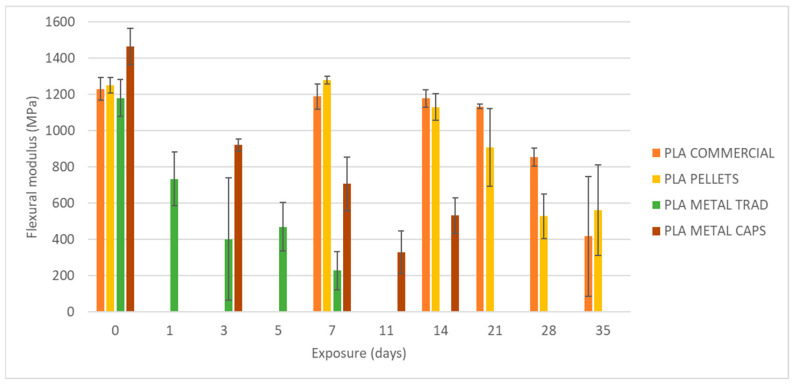
Variation of the elastic flexural modulus with exposure to basic pH and temperature.

**Figure 16:**
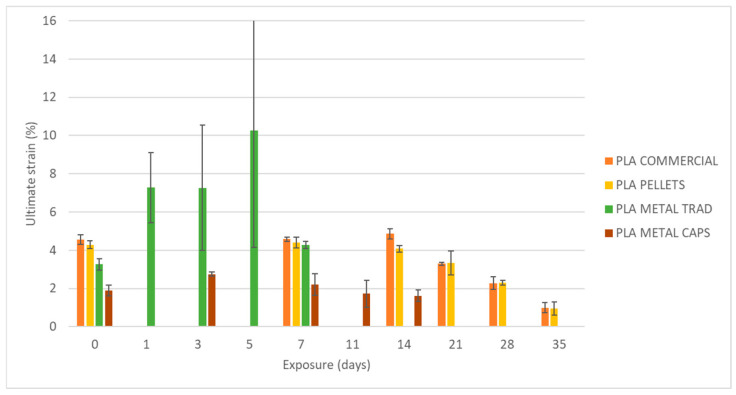
Variation of strain at break with exposure to basic pH and temperature.

Figure 14, Figure 15 and Figure 16 present the evolution of the flexural strength, the elastic flexural modulus, and the flexural strain at break of specimens exposed to a basic pH similar to that of fresh concrete.

Firstly, it is observed that PLA without additives (commercial PLA and PLA made from pellets) shows a lower degradation rate compared to formulations containing metal powder. This behavior can be attributed to the higher susceptibility of the metal (iron and its alloys) to oxidation, which would accelerate its deterioration in alkaline media. As a consequence, the exposure times initially foreseen (35 days) had to be reduced for the metal-containing materials, being limited to 11 days for PLA+metal manufactured by direct mixing and 21 days for PLA+metal obtained by encapsulation. In addition, the specimens that reached these exposure periods could not be tested due to the drastic reduction in their dimensions, which prevented the testing machine from applying the minimum required load. For this reason, flexural strength, elastic flexural modulus, and the flexural strain at break values for all exposure times are not presented in the figures.

Analysis of the results reveals a reduction of up to 82% in the flexural strength of the commercial PLA after 35 days of exposure from 37 MPa to 6.87 MPa. An identical decrease (82%) is recorded for PLA made from pellets, although in this case, the material experiences a slight increase of about 4 MPa in its flexural strength after one week of exposure, before starting its progressive degradation. In addition, some stability in flexural strength, strain at break, and elastic modulus is observed during the first two weeks of exposure, followed by an acceleration of mechanical deterioration.

In the case of PLA with metal additives, the degradation of mechanical properties is more pronounced in the specimens manufactured by direct mixing with metal powder (PLA_met_trad). In addition, this method shows a higher dispersion of results, especially in terms of deformation, indicating a lower reproducibility and an accelerated degradation of the material. These findings suggest that fabrication by direct mixing is not the most suitable method, as it leads to a significant loss of uniformity and reliability in the mechanical properties of the material.

On the other hand, the specimens obtained by encapsulation were able to remain submerged for 10 days longer than those manufactured by direct mixing. However, they also suffered severe deterioration, with a 65% reduction in flexural strength after 14 days of exposure and complete degradation (100%) at 21 days.

**Figure 17:**
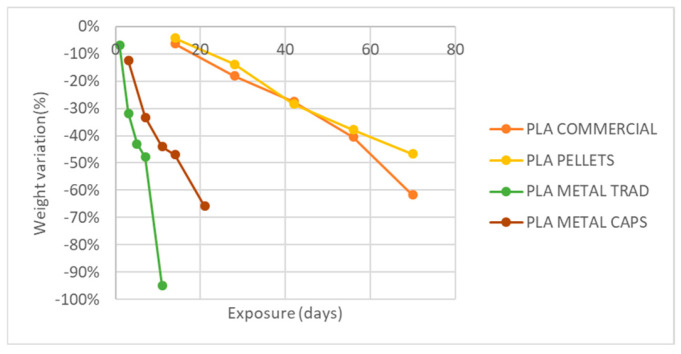
Variation of specimen weight with exposure to basic pH and temperature.

**Figure 18:**
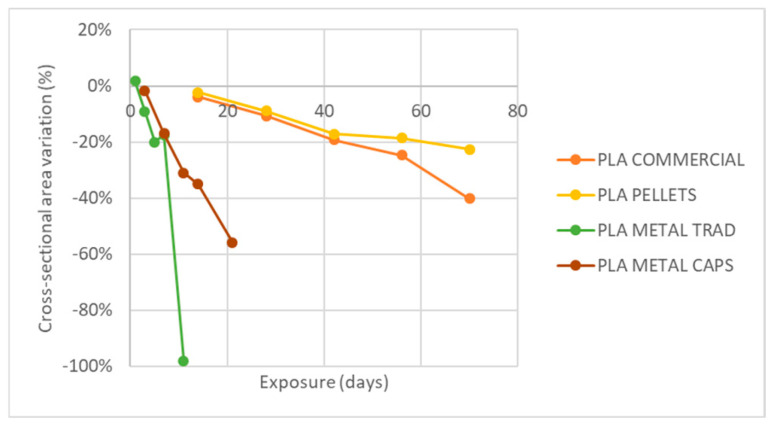
Variation of specimen cross-section with exposure to basic pH and temperature.

The analysis of weight reduction and the cross-section (Figure 17 and Figure 18) reveals that pure PLA, both in its commercial version and in the one obtained from pellets, shows an approximately linear decrease in both parameters. In the case of commercial PLA, the weight reduction reaches up to 62%, while the cross-section decreases by 40% after 70 days of exposure. However, a more pronounced degradation is observed in the commercial PLA during the last two extractions compared to the PLA made from pellets during the same periods.

On the other hand, PLA specimens with metal addition exhibit a considerably faster weight loss than in the previous cases. In specimens made by encapsulation, the weight reduction amounts to 65% in only 21 days, while in those obtained by direct mixing of pellets with metal powder in the hopper (trad method), the weight loss reaches almost 95% in only 11 days.

Finally, as expected, the ANOVA results confirmed that all evaluated parameters exhibit statistically significant variation under alkaline pH exposure, with *p*-values equal to 0 in all cases. This extremely low *p*-value is attributable to the clear and consistent differences observed between the mean values of the groups corresponding to each exposure time.

### 4.4. Geometric Changes After Degradation

**Figure 19:**
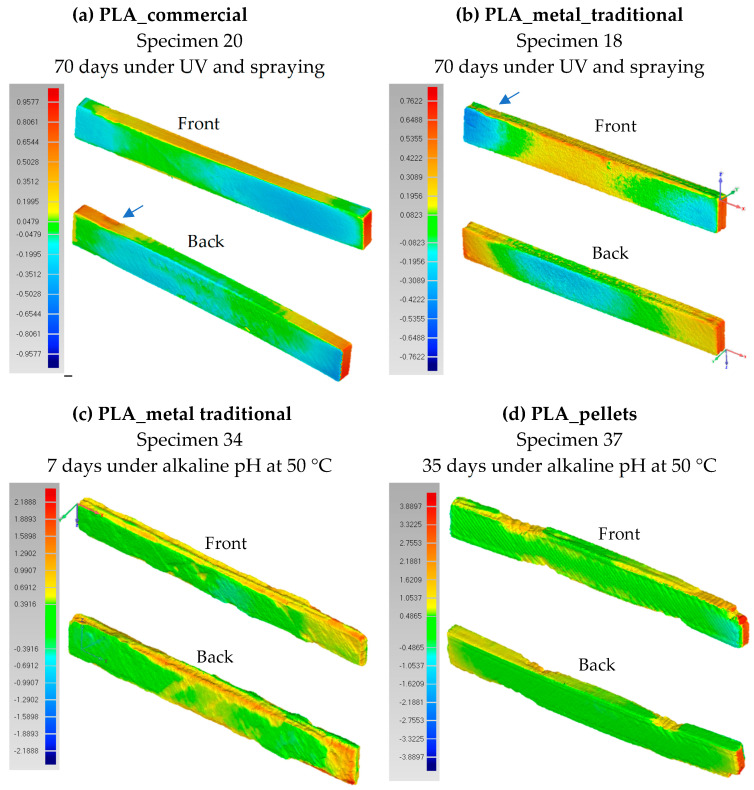
Dimensional deviations of (**a**) commercial PLA after 70 days under UV and spraying, (**b**) PLA with metal (traditional) after 70 days under UV and spraying, (**c**) PLA with metal (traditional) after 7 days under alkaline pH at 50 °C, and (**d**) PLA from pellets after 35 days under alkaline pH at 50 °C.

A 3D scanner was used to compare the dimensions of the sample before and after exposure (Figure 19). In scans a and b, the behavior of commercial PLA and PLA with traditional metal fillers after 70 days of UV exposure and spraying is analyzed. In both cases, a constriction mark is visible on the left side of the specimen, caused by the clamp used to secure the samples to the UV chamber grid. Although this might suggest a slight increase in the cross-sectional area, Figure 12 confirms that this increase is less than 0.2% or even negligible in the case of PLA with traditional metal fillers. Therefore, this mark is attributed to the compression of the four corners due to the softening of the material under UV radiation exposure. Additionally, both specimens (Figure 19a and b) exhibit warping, likely resulting from the combined effects of temperature and humidity. This deformation is more pronounced in the case of PLA with traditional metal fillers. The greatest dimensional deviations compared to the pre-exposure specimen are observed at the edges; however, they do not exceed 0.96 mm for commercial PLA and 0.77 mm for PLA with traditional metal fillers.

Scans c and d (Figure 19) illustrate the behavior of PLA with traditional metal fillers and PLA with pellet-based fabrication after exposure to an alkaline pH environment at 50 °C. A significant difference in geometric degradation is observed, with the traditionally manufactured PLA–metal specimens exhibiting greater deterioration compared to those produced from pellets, even considering the difference in exposure time (7 days for PLA_metal_trad versus 35 days for PLA_pellets). Focusing first on the PLA_pellets specimen, three distinct erosion zones can be identified: two on one side and two more on the opposite side, one of which is located at a corner. These defects may be attributed to printing errors that were not detected prior to exposure, leading to an increased number of voids that facilitated solution penetration and accelerated degradation. However, these are localized defects that could be mitigated by improving print quality. The greatest deviation from the original, non-degraded geometry occurs along the specimen’s length, with the maximum displacement observed at the corner, reaching a magnitude of 3.9 mm.

**Figure 20:**
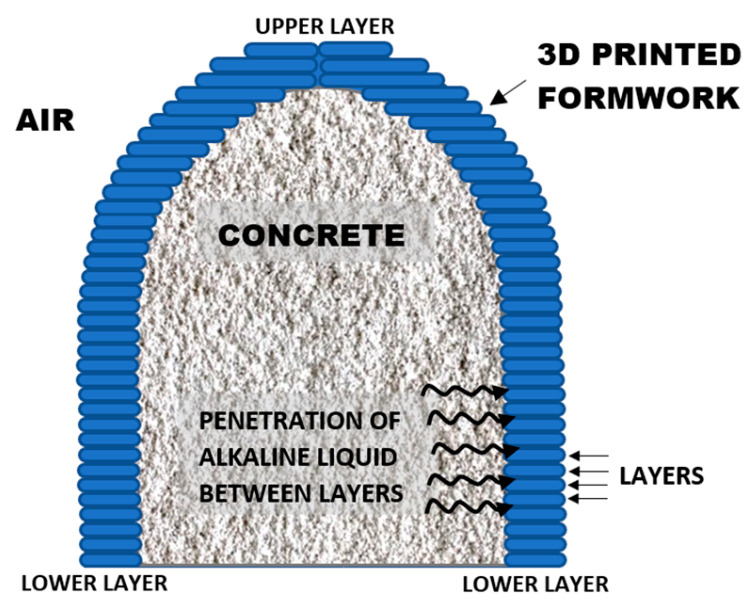
Schematic representation of the contact interface between the external 3D-printed polymer formwork and the concrete during an artificial reef construction, illustrating the layered structure of the formwork and the potential penetration of alkaline liquid between layers.

Conversely, in the traditionally manufactured PLA–metal specimens (c), a different degradation mechanism occurs. The results indicate the presence of areas of material loss in the contours of some of the intermediate layers of the specimen, suggesting that the basic pH solution may have penetrated into the interior of the structure, causing weight loss and delamination. Figure 19c shows these voids in the interlayers from two different orientations. Extrapolating this behavior to the manufacturing process of real concrete parts, such as artificial reefs, like those in Figure 1, and of other concrete types, like columns or sculptures [5,6,7], the PLA formwork would be subjected to direct exposure to a basic pH on its outer wall, specifically at the layer boundaries (Figure 20), rather than on the flat surfaces of the top and bottom layers. As demonstrated in the scan, this zone exposed to an alkaline pH is highly susceptible to formwork failure. The presence of voids—whether due to porosity, material deficiencies, or poor interlayer adhesion—can allow the alkaline liquid mortar to infiltrate, progressively degrading the specimen from within. This deterioration reduces the load-bearing capacity of the formwork and, in the worst case, can lead to interlayer delamination and structural failure, causing the formwork to break into multiple parts.

The results of PLA_metal_trad under alkaline pH at 50 °C also show an uneven degradation, with areas of high material disappearance, especially in the right and central part, while the left part shows less loss. This heterogeneity in degradation makes it very difficult to accurately determine the actual cross-section at fracture during the flexural test.

### 4.5. Equivalence to Real-Time Exposure

To establish an equivalence between the experimental exposure time and real-world exposure, the following considerations were considered:

♦ The weathering exposure equipment does not accelerate the UV effect of sunlight but rather simulates it. The accelerated aging effect is due to continuous operation, including night-time exposure, rather than harsher conditions.
♦ Two locations in Spain with distinct climatic conditions were selected: Cantabria, characterized by higher rainfall and fewer hours of solar exposure, and Seville, which has a drier climate with significantly greater solar exposure. According to the solar panel company Solfy [33], the annual direct solar exposure in Cantabria is 1639 h, whereas in Seville, it reaches 3526 h. Regarding precipitation, Cantabria records an annual average of 1095.6 mm, whereas Seville experiences considerably lower levels, at 336 mm [34]. Therefore, the lifespan of the plastic when exposed to atmospheric agents varies depending on the geographic location.

Based on these data and considering that the equipment’s solar and rain cycles consist of 5 h of solar exposure followed by 1 h of rainfall, the total equivalent real-world exposure time (in months) for these two geographic regions was estimated. This analysis allowed for the evaluation of variations in the mechanical properties of maximum tensile strength and strain at break compared to the non-degraded material (Table 6).

Regarding the equivalence with spraying, no relevant references have been found to establish a direct comparison with real-world conditions. A possible quantitative analysis could be conducted as follows: each panel, equipped with three nozzles, sprays 1.75 L/min over an area of 280 mm × 485 mm. Consequently, each specimen, with a surface area of 10 mm × 80 mm, receives 0.618 L/h. In Cantabria, the average annual precipitation is 1095 L/m^2^, which corresponds to 0.876 L per specimen per year. This implies that one hour of testing in the chamber would subject the specimens to an amount of water equivalent to 70% of the total annual precipitation in Cantabria. In Seville, where annual precipitation amounts to 336 L/m^2^, one hour of testing in the chamber would expose each specimen to 2.3 times the amount of rainfall received in an entire year under natural conditions.

However, a strictly quantitative evaluation is not considered appropriate, as the alternation between one hour of water spraying and four hours of UV exposure induces a temperature fluctuation from 50 °C (under UV radiation) to 25 °C during water spraying and relevant variations in surface humidity on the specimens, which are complex to quantify and equate to real environmental conditions.

By analyzing Table 6, the most significant variations in maximum flexural strength and strain at break occur after 56 days of experimentation in PLA–metal specimens, with a more pronounced effect on maximum strain. This corresponds to 8.3 months of solar exposure in Cantabria or 3.9 months in Seville. However, for PLA specimens without metal, a critical level of deterioration may be expected after 70 days of testing, which equates to 10.4 months in Cantabria or 4.8 months in Seville.

Regarding the equivalence between the pH exposure test and real-world conditions, formworks for structures, such as the artificial reefs shown in Figure 1, with an approximate volume of 300 dm^3^, are considered. These structures were demolded one day after the mortar was poured, representing the period during which the high-pH environment actively contributed to the degradation of the formwork. Therefore, the fabrication of a single reef corresponds to approximately 24 h of exposure to alkaline conditions. Under this hypothesis, establishing a correlation between the experimental exposure time and real-world conditions is straightforward, as one day of exposure in the experiment is considered equivalent to one day in actual conditions (Table 7). Although the concrete gradually hardens over this period, leading to a reduction in moisture content, accurately determining the precise moment at which its alkaline pH no longer affects the polymer remains highly complex. Consequently, it is assumed that the material undergoes continuous exposure to alkaline degradation throughout the entire contact period. This approach ensures a conservative and safety-oriented assessment.

Based on this hypothesis, it can be concluded that after the fabrication of 28 components, corresponding to 28 days of exposure, the formworks manufactured from commercial PLA and PLA derived from pellets exhibit a reduction of 54% and 69%, respectively, in their load-bearing capacity, along with a 50% decrease in strain at break.

Notably, the PLA_met_trad formulation demonstrates a distinct mechanical response. After just three days of use, it undergoes a 62% reduction in flexural strength, while from the first day, its maximum strain increases by up to 123%. This pronounced increase in deformability is attributed to the weaker interlayer adhesion between printed layers, which promotes interlayer slippage, enabling greater bending under reduced loads.

In contrast, the behavior of PLA_met_caps falls between the previously analyzed materials, although its durability remains significantly lower than that of unmodified PLA. Specifically, when incorporating metal encapsulation, the formwork fabricated from this material experiences a 50% reduction in flexural strength after seven reuse cycles.

In particular, for the initially intended application—namely, the fabrication of artificial reefs or any other ornamental structure—the significance of the mechanical degradation tests can be interpreted as follows. If the formwork design is carried out using finite element analysis software, such as Ansys or Abaqus, it is standard engineering practice to apply a safety factor to the design loads. Assuming a safety factor of 1.2 applied to the loads, a 20% reduction in flexural strength due to material degradation would mark the threshold at which the structural integrity of the formwork is compromised. Therefore, setting a usability limit at a 20% decrease in flexural strength, it can be estimated that PLA_commercial formworks could be used for up to approximately 21 days, while PLA_pellet formworks would be suitable for around 18 days. The incorporation of metallic fillers does not appear to provide any benefit in terms of durability under these conditions; instead, it accelerates degradation, with the 20% reduction being reached in as little as 1 to 3 days, depending on whether PLA_metal_trad or PLA_metal_caps is used, respectively. These estimations are based on the most aggressive exposure condition tested, namely, immersion in an alkaline environment.

### 4.6. Applicability to Artificial Reef (AR) Manufacturing

This research contributes to the expanding field of sustainable marine restoration through the development of innovative manufacturing strategies for artificial reefs. Among these, 3D printing has emerged as a particularly promising technique. The review by Yoris-Nobile et al. [35] systematizes current practices in the design, material selection, and fabrication of artificial reefs via additive manufacturing. Most existing approaches rely on the direct 3D printing of reef structures using the extrusion of cementitious or geopolymer mortars, which introduces challenges related to rheological control and the mechanical stability of the printed elements. In particular, Yoris-Nobile et al. [36] emphasize the need for a carefully balanced mortar formulation—neither overly fluid nor excessively stiff—to ensure both geometric fidelity during printing and sufficient mechanical resistance to withstand marine forces, such as waves and currents.

The comprehensive review by Matus et al. [37] further explores additive manufacturing methods applied to marine restoration, including binder jetting, polymer extrusion (FFF), and the use of cementitious or geopolymer materials. While polymer-based approaches are noted for their environmental limitations and low durability in seawater, cement-based systems are recognized for their structural robustness. However, all reviewed cases rely on the direct 3D printing of the final reef geometry. None consider the use of polymeric molds created via 3D printing and subsequently filled with a mortar or geopolymer, highlighting the originality of the method proposed in this study.

Additionally, the work by Silva Lima et al. [38] provides a broad overview of artificial reef research, covering ecological, engineering, socio-economic, and environmental aspects. Although not focused specifically on additive manufacturing, this review underscores the importance of integrating ecological functionality with fabrication efficiency—an objective that aligns with the methodology presented here, which enables controlled production of complex reef geometries while maintaining structural integrity.

The method developed in this study, based on 3D-printed polymer molds filled with conventional cementitious mixtures, offers notable advantages over direct extrusion printing. These include lower sensitivity to environmental conditions during manufacturing, the use of standard mortar formulations without rheological modification, and the potential for mold reuse, improving both sustainability and economic viability. To date, five artificial reefs have been fabricated using this technique with PLA pellet-based formworks, with the aim of deploying them in the Bay of Santander (Spain). These units will serve to compare biodiversity outcomes with those from reefs produced via direct mortar printing, following a defined immersion period of 1 year. Although biodiversity data are not yet available, this field deployment seeks to validate the capacity of the proposed solution to foster ecological enhancement in marine environments. From a manufacturing standpoint, the molds performed reliably; no degradation-related damage was observed, and the system effectively withstood all applied loads and boundary conditions. Therefore, this study has already confirmed the technical feasibility of using PLA for the production of reusable molds, offering a robust, low-cost, and adaptable solution for fabricating complex cementitious structures for marine applications.

While the scope of this research extends beyond AR manufacturing, the proposed approach is particularly well suited to such applications. It enables the replication of biologically inspired, complex geometries essential for promoting marine biodiversity in a controlled and potentially scalable manner. Compared to existing methods, this hybrid technique—combining 3D-printed molds with traditional casting—constitutes a versatile and innovative contribution to ecological restoration in coastal and marine environments.

## 5. Conclusions

In this study, the behavior of four types of 3D-printed specimens, manufactured using different combinations of materials and production methods, was analyzed in order to evaluate the effect of exposure to environmental conditions of sun and rain, as well as alkaline solutions, on their degradation. For this purpose, three-point bending tests were carried out, and the dimensions and weights of the specimens before and after exposure were analyzed.

From the results obtained, the following conclusions can be drawn:

♦ Exposure to an alkaline medium at 50 °C is significantly more aggressive than exposure to UVA radiation and spray. This aspect is crucial in the manufacture of formwork with PLA or PLA with metal additives, as these materials have a limited lifetime before degradation, leading to a loss of both strength and dimensions.
♦ The dispersion of flexural strength, elastic modulus, and strain at break values increases with exposure time. Also, the addition of metal powder increases the variability of the results in both formulations evaluated. This suggests that the printing process plays a fundamental role in the degradation of the material since the presence of pores or micro-defects in the specimens facilitates the penetration of liquids (distilled water from the spray or alkaline solutions), which modifies both the mechanism and the rate of degradation.
♦ The incorporation of 50% by weight of metal powder into PLA not only fails to improve its flexural mechanical properties but actually results in their significant deterioration under alkaline pH conditions. Specifically, a reduction in flexural strength of approximately 65% is observed after 7 days of exposure when the PLA pellets are directly mixed with metal powder, and after 14 days when the filament is produced using pre-filled capsules. Therefore, PLA with metallic additives is not suitable for formwork applications. In contrast, virgin PLA—whether in filament or pellet form—remains stable for up to 14 days of exposure, after which a progressive decline in flexural strength occurs, reaching a reduction of approximately 82% after 35 days. Consequently, while the addition of metal powder is not recommended, virgin PLA emerges as a more reliable alternative, allowing for the reuse of the same formwork for at least 14 days (or 14 casting cycles) with only a 6% decrease in flexural strength.
♦ The most suitable material for use as concrete formwork is unmodified PLA. There are no conclusive results regarding whether commercial PLA or PLA with pellet additives performs better. No significant differences were found in the maximum flexural strength and elastic modulus between the specimens made from commercial PLA in coil and those obtained from pellets in the laboratory. In terms of ultimate strain, commercial PLA specimens show slightly higher values (between 4% and 30%), although these differences disappear after 70 days of exposure to UVA radiation and spraying, as well as after 35 days of exposure to the alkaline solution. Therefore, it can be determined that both exhibit nearly identical behavior. This confirms that the addition of colorant pellets during the manufacturing of commercial PLA does not introduce any plasticizers that enhance its properties. If any plasticizing effect is present, its proportion is either negligible or insufficient.
♦ While the degradation of commercial PLA and pellet-based PLA in an alkaline pH environment leads to localized material loss in highly concentrated areas, the dissolution attack on PLA–metal specimens (in both manufacturing processes studied) induces delamination, i.e., interlayer separation, occurring unevenly along the entire length of the specimen.
♦ The exposure of 3D-printed PLA beyond 8.3 months in humid climates with lower solar exposure (e.g., Northern Spain) or 3.9 months in dry, high-sunlight environments (e.g., Southern Spain) is not recommended, especially if PLA with metal powder additions is employed. The determination of the exposure limit to alkaline pH mortar, beyond which the use of formworks should be discontinued, depends on the specific application and, in particular, on the safety factor considered in their design to withstand hydrostatic pressure, mold tightening, and demolding forces. Table 6 provides an estimate of the variation in mechanical resistance, offering a quantitative basis for decision making according to the selected safety factor.

The degradation results obtained under atmospheric conditions of UVA radiation and spraying are not only applicable to the use of formworks in outdoor environments but also to any other 3D-printed components with the same composition that are exposed to sunlight and rainfall. Examples include protective casings for outdoor electronic devices, structural elements in temporary architectural installations, and customized components for urban furniture.

Although the present work focuses on the evaluation of mechanical performance and dimensional stability of PLA-based materials under alkaline and outdoor exposure, further research is required to understand the underlying molecular degradation mechanisms. Future studies should include advanced analytical techniques, such as Gel Permeation Chromatography (GPC), to determine changes in molecular weight and estimate degradation rates. Additionally, Differential Scanning Calorimetry (DSC) and Fourier-Transform Infrared (FTIR) spectroscopy can provide insights into thermal transitions and structural modifications of the polymer chains, respectively. Incorporating these techniques would complement the findings of this study and contribute to a more comprehensive understanding of the degradation behavior of these materials under realistic service conditions.

## Figures and Tables

**Table 1:** Composition of the metal scale.

	Percentage
CaO	0.07
CoO	0.009
Cr_2_O_3_	0.15
CuO	0.147
Phases Fe	96.7
MnO	0.733
MoO_3_	0.029
NiO	0.06
PbO	0.003
SO_3_	0.05
SiO_2_	0.24
SnO_2_	0.01
WO_3_	0.003
ZnO	0.056

**Table 2:** Samples in each case study. A total of 45 samples are printed in each material.

	Atmospheric Conditions	Basic PH	No Degradation
Group 1	Samples from 1 to 4	Samples from 21 to 24	
Group 2	Samples from 5 to 8	Samples from 25 to 28	
Group 3	Samples from 9 to 12	Samples from 29 to 32	Samples from 41 to 45
Group 4	Samples from 13 to 16	Samples from 33 to 36	
Group 5	Samples from 17 to 20	Samples from 37 to 40	

**Table 3:** Extrusion temperatures. T1 is located closest to the hopper, whilst T4 is the resistance closest to the exit.

	T_1_	T_2_	T_3_	T_4_
1_PLAco	170	185	190	170
2_PLApe	170	185	190	170
3_PLAmet_tra	195	190	200	180
4_PLAmet_caps	195	190	190	175

**Table 4:** Printing parameters.

	Flexural Testing Specimens	Capsules
Nozzle diameter	0.8 mm	0.4 mm
Printing temperature	210 °C	210 °C
Bed temperature	60 °C	60 °C
Wall lines	2	2
Printing speed	40 mm/s	40 mm/s

**Table 5:** *p*-values and significance assessment for the five parameters evaluated in each of the four case studies.

Case Study	Parameter	*p*-Value	Are There Statistically Significant Variations?
PLA_commercial	Flexural strength (MPa)	0.156	NO
	Flexural modulus (MPa)	0.147	NO
	Ultimate strain (-)	0.014	SI
	Section variation (%)	0.351	NO
	C-S area variation (%)	0.007	SI
PLA_pellets	Flexural strength (MPa)	0.255	NO
	Flexural modulus (MPa)	0.104	NO
	Ultimate strain (-)	0.020	SI
	Section variation (%)	0.332	NO
	C-S area variation (%)	0.701	NO
PLA_met_trad	Flexural strength (MPa)	0.294	NO
	Flexural modulus (MPa)	0.420	NO
	Ultimate strain (-)	0.001	SI
	Section variation (%)	0.041	SI
	C-S area variation (%)	0.014	SI
PLA_met_caps	Flexural strength (MPa)	0.159	NO
	Flexural modulus (MPa)	0.088	NO
	Ultimate strain (-)	0.048	SI
	Section variation (%)	0.006	SI
	C-S area variation (%)	0.202	NO

**Table 6:** Equivalence of laboratory degradation and real-time degradation related to atmosphere conditions of UV and spraying.

Exposure Time in the Test (Days)	Equivalence of Sun Exposure in Real Time (Months)		PLA_co		PLA_pe		PLA_met_trad		PLA_met_caps	
Experiment	Cantabria	Seville	${{∆\sigma }_{f}}_{max}$ (%)	${{∆\varepsilon }_{f}}_{max}$ (%)	${{∆\sigma }_{f}}_{max}$ (%)	${{∆\varepsilon }_{f}}_{max}$ (%)	${{∆\sigma }_{f}}_{max}$ (%)	${{∆\varepsilon }_{f}}_{max}$ (%)	${{∆\sigma }_{f}}_{max}$ (%)	${{∆\varepsilon }_{f}}_{max}$ (%)
14	2.1	1.0	8.5	−7.1	5.5	−9.3	−6.4	−8.2	11.2	−2.5
28	4.2	1.9	−14.7	−25.0	1.8	−19.2	4.3	−2.1	−2.1	−23.9
42	6.2	2.9	5.8	−21.5	8.8	−31.7	−13.8	−13.2	6.0	1.4
56	8.3	3.9	1.1	−7.3	−1.3	−32.8	−16.2	−43.8	−37.8	−44.8
70	10.4	4.8	−28.6	−43.2	−3.9	−42.6	−2.6	−9.3	−19.9	−29.3

${{∆\sigma }_{f}}_{max}$ is the variation of flexural strength and ${{∆\varepsilon }_{f}}_{max}$ is the variation of flexural strain at break.

**Table 7:** Equivalence of laboratory degradation and real-time degradation related to exposure to basic pH at 50 °C.

Exposure Time in the Test (Days)	Equivalence in Real Pieces Manufactured (nº Pieces)	PLA_co		PLA_pe		PLA_met_trad		PLA_met_caps	
		${{∆\sigma }_{f}}_{max}$ (%)	${{∆\varepsilon }_{f}}_{max}$ (%)	${{∆\sigma }_{f}}_{max}$ (%)	${{∆\varepsilon }_{f}}_{max}$ (%)	${{∆\sigma }_{f}}_{max}$ (%)	${{∆\varepsilon }_{f}}_{max}$ (%)	${{∆\sigma }_{f}}_{max}$ (%)	${{∆\varepsilon }_{f}}_{max}$
1	1	-	-	-	-	−23.5	122.7	-	-
3	3	-	-	-	-	−61.6	122.4	−21.5	44.6
5	5	-	-	-	-	−63.5	214.6	-	-
7	7	−3.2	0.4	9.2	2.9	−64.3	30.8	−49.4	16.5
11	11	-	-	-	-	−100	−100	−62.8	−9.0
14	14	−6.4	6.7	−6.2	−4.9	-	-	−65.3	−14.3
21	21	−18.3	−27.7	−32.8	−22.3	-	-	−100	−100
28	28	−53.7	−50.0	−68.8	−46.3	-	-	-	-
35	35	−81.6	−78.2	−81.9	−77.7	-	-	-	-

## Data Availability

Dataset available on request from the authors.

## References

[1] Mohanavel V., Ashraff Ali K.S., Ranganathan K., Allen Jeffrey J., Ravikumar M.M., Rajkumar S. (2021). The Roles and Applications of Additive Manufacturing in the Aerospace and Automobile Sector. Mater. Today Proc..

[2] Dunham S., Mosadegh B., Romito E.A., Zgaren M. (2018). Applications of 3D Printing. 3D Printing Applications in Cardiovascular Medicine.

[3] Gomes Correia V.M., Pereira N., Perinka N., Costa P., del Campo J., Lanceros-Mendez S. (2022). Printed 3D Gesture Recognition Thermoformed Half Sphere Compatible with In-Mold Electronic Applications. Adv. Eng. Mater..

[4] Sivakumar N.K., Palaniyappan S., Vishal K., Alibrahim K.A., Alodhayb A., A M.K. (2024). Crushing Behavior Optimization of Octagonal Lattice-structured Thin-walled 3D-Printed Carbon Fiber Reinforced PETG (CF/PETG) Composite Tubes under Axial Loading. Polym. Compos..

[5] Jipa A., Giacomarra F., Giesecke R., Chousou G., Pacher M., Dillenburger B., Lomaglio M., Leschok M. (2019). 3D-Printed Formwork for Bespoke Concrete Stairs. Proceedings of the ACM Symposium on Computational Fabrication.

[6] Lloret-Fritschi E., Wangler T., Gebhard L., Mata-Falcón J., Mantellato S., Scotto F., Burger J., Szabo A., Ruffray N., Reiter L. (2020). From Smart Dynamic Casting to a Growing Family of Digital Casting Systems. Cem. Concr. Res..

[7] Jipa A., Dillenburger B. (2022). 3D Printed Formwork for Concrete: State-of-the-Art. Opportunities. Challenges. and Applications. 3D Print. Addit. Manuf..

[8] Korniejenko K., Gądek S., Dynowski P., Tran D.H., Rudziewicz M., Pose S., Grab T. (2024). Additive Manufacturing in Underwater Applications. Appl. Sci..

[9] Talekar S., Barrow C.J., Nguyen H.C., Zolfagharian A., Zare S., Farjana S.H., Macreadie P.I., Ashraf M., Trevathan-Tackett S.M. (2024). Using waste biomass to produce 3D-printed artificial biodegradable structures for coastal ecosystem restoration. Sci. Total Environ..

[10] Jipa A., Bernhard M., Dillenburger B. (2017). Submillimetre Formwork: 3D-Printed Plastic Formwork for Concrete Elements. 2017 TxA Emerging Design + Technology Conference Proceedings.

[11] Jipa A., Reiter L., Flatt R.J., Dillenburger B. (2022). Environmental Stress Cracking of 3D-Printed Polymers Exposed to Concrete. Addit. Manuf..

[12] Csótár H., Szalai S., Kurhan D., Sysyn M., Fischer S. (2025). Evaluating 3D-Printed Polylactic Acid (PLA)-Reinforced Materials: Mechanical Performance and Chemical Stability in Concrete Mediums. Appl. Sci..

[13] Zaaba N.F., Jaafar M. (2020). A Review on Degradation Mechanisms of Polylactic Acid: Hydrolytic. Photodegradative. Microbial. and Enzymatic Degradation. Polym. Eng. Sci..

[14] Cuiffo M.A., Snyder J., Elliott A.M., Romero N., Kannan S., Halada G.P. (2017). Impact of the Fused Deposition (FDM) Printing Process on Polylactic Acid (PLA) Chemistry and Structure. Appl. Sci..

[15] Lee S., Wee J.W. (2024). Effect of temperature and relative humidity on hydrolytic degradation of additively manufactured PLA: Characterization and artificial neural network modeling. Polym. Degrad. Stab..

[16] Valerga Puerta A.P., Fernandez-Sanz G., Bañon F., Fernandez-Vidal S.R. (2023). Biodegradable materials with FDM technology under the aging effect of solar and saltwater exposure. Adv. Mech. Eng..

[17] Sedlak J., Joska Z., Jansky J., Zouhar J., Kolomy S., Slany M., Svasta A., Jirousek J. (2023). Analysis of the Mechanical Properties of 3D-Printed Plastic Samples Subjected to Selected Degradation Effects. Materials.

[18] Schweizer K., Bhandari S., Lopez-Anido R.A., Korey M., Tekinalp H. (2024). Recycling Large-Format 3D Printed Polymer Composite Formworks Used for Casting Precast Concrete—Technical Feasibility and Challenges. J. Compos. Constr..

[19] Emami N. (2024). Additive Manufacturing of TPU and Hybrid TPU-PLA Formwork for Custom Repetitive Precast Concrete. J. Archit. Eng..

[20] Castanon-Jano L., Palomera-Obregon P., Blanco-Fernandez E., Indacoechea-Vega I. (2023). Analysis of Manufacturing and Material Parameters in 3D-Printed Polylactic Acid (PLA) Parts Filled with Glass Powder: Mechanical. Economic. and Environmental Assessment. Int. J. Adv. Manuf. Technol..

[21] Castanon-Jano L., Palomera-Obregon P., Lázaro M., Blanco-Fernandez E., Blasón S. (2024). Enhancing Sustainability in Polymer 3D Printing via Fusion Filament Fabrication through Integration of By-Products in Powder Form: Mechanical and Thermal Characterization. Int. J. Adv. Manuf. Technol..

[22] Darsin M., Sabariman W.A., Trifiananto M., Fachri B.A. (2023). Flexural Properties of Metal 3D Printing Products Using PLA-Stainless Steel Filament. AIP Conference Proceedings.

[23] Tao Y., Wang H., Li Z., Li P., Shi S.Q. (2017). Development and Application of Wood Flour-Filled Polylactic Acid Composite Filament for 3D Printing. Materials.

[24] Razali M.S., Khimeche K., Melouki R., Boudjellal A., Vroman I., Alix S., Ramdani N. (2022). Preparation and Properties Enhancement of Poly(Lactic Acid)/Calcined-Seashell Biocomposites for 3D Printing Applications. J. Appl. Polym. Sci..

[25] Kariz M., Sernek M., Kuzman M.K. (2018). Effect of Humidity on 3D-Printed Specimens from Wood-PLA Filaments. Wood Res..

[26] Figueroa-Velarde V., Diaz-Vidal T., Cisneros-López E.O., Robledo-Ortiz J.R., López-Naranjo E.J., Ortega-Gudiño P., Rosales-Rivera L.C. (2021). Mechanical and Physicochemical Properties of 3D-Printed Agave Fibers/Poly(lactic) Acid Biocomposites. Materials.

[27] Ramezani R., Alizadeh R., Labbaf S. (2025). 3D-printed PLA/Fe3O4/MgO hybrid composite scaffolds with improved properties. Bioprinting.

[28] Díaz-García Á., Law J.Y., Cota A., Bellido-Correa A., Ramírez-Rico J., Schäfer R., Franco V. (2020). Novel Procedure for Laboratory Scale Production of Composite Functional Filaments for Additive Manufacturing. Mater. Today Commun..

[29] Díaz-García Á., Law J.Y., Felix M., Guerrero A., Franco V. (2022). Functional. Thermal and Rheological Properties of Polymer-Based Magnetic Composite Filaments for Additive Manufacturing. Mater. Des..

[30] (2016). Plásticos. Métodos de Exposición a Fuentes Luminosas de Laboratorio.

[31] Li S., McCarthy S. (1999). Further investigations on the hydrolytic degradation of poly (DL-lactide). Biomaterials.

[32] (2020). Plásticos. Determinación de Las Propiedades de Flexión.

[33] Rodríguez Buñuel S. Mapa de Radiación Solar En España: Tablas Por Provincias. https://solfy.net/placas-solares/mapa-de-radiacion-solar-en-espana-tablas-por-provincias/.

[34] Instituto Nacional de Estadística (2016). Boletín Mensual de Estadística. Climatología.

[35] Yoris-Nobile A.I., Slebi-Acevedo C.J., Lizasoain-Arteaga E., Indacoechea-Vega I., Blanco-Fernandez E., Castro-Fresno D., Alonso-Estebanez A., Alonso-Cañon S., Real-Gutierrez C., Boukhelf F. (2023). Artificial reefs built by 3D printing: Systematisation in the design, material selection and fabrication. Constr. Build. Mater..

[36] Yoris-Nobile A.I., Lizasoain-Arteaga E., Slebi-Acevedo C.J., Blanco-Fernandez E., Alonso-Cañon S., Indacoechea-Vega I., Castro-Fresno D. (2023). Life cycle assessment (LCA) and multi-criteria decision-making (MCDM) analysis to determine the performance of 3D printed cement mortars and geopolymers. J. Sustain. Cem.-Based Mater..

[37] Matus I.V., Alves J.L., Góis J., Vaz-Pires P., Barata da Rocha A. (2024). Artificial reefs through additive manufacturing: A review of their design, purposes and fabrication process for marine restoration and management. Rapid Prototyp. J..

[38] Silva Lima J., Rosental Zalmon I., Love M. (2019). Overview and trends of ecological and socioeconomic research on artificial reefs. Mar. Environ. Res..

